# Genetics: A Starting Point for the Prevention and the Treatment of Obesity

**DOI:** 10.3390/nu15122782

**Published:** 2023-06-17

**Authors:** Giuseppe Novelli, Carmen Cassadonte, Paolo Sbraccia, Michela Biancolella

**Affiliations:** 1 Department of Biomedicine and Prevention, Medical School, University of Rome Tor Vergata, Via Montpellier 1, 00133 Rome, Italy; carmen.cassadonte@students.uniroma2.eu; 2 Italian Barometer Diabetes Observatory Foundation, IBDO, 00186 Rome, Italy; sbraccia@med.uniroma2.it; 3 Department of Pharmacology, School of Medicine, University of Nevada, Reno, NV 89557, USA; 4 Department of Systems Medicine, Medical School, University of Rome Tor Vergata, Via Montpellier 1, 00133 Rome, Italy; 5 Department of Biology, Tor Vergata University of Rome, Via della Ricerca Scientifica 1, 00133 Rome, Italy; michela.biancolella@uniroma2.it

**Keywords:** obesity, genetics, epigenetics, mutations, polygenic score

## Abstract

Obesity is a common, serious, and costly disease. More than 1 billion people worldwide are obese—650 million adults, 340 million adolescents, and 39 million children. The WHO estimates that, by 2025, approximately 167 million people—adults and children—will become less healthy because they are overweight or obese. Obesity-related conditions include heart disease, stroke, type 2 diabetes, and certain types of cancer. These are among the leading causes of preventable, premature death. The estimated annual medical cost of obesity in the United States was nearly $173 billion in 2019 dollars. Obesity is considered the result of a complex interaction between genes and the environment. Both genes and the environment change in different populations. In fact, the prevalence changes as the result of eating habits, lifestyle, and expression of genes coding for factors involved in the regulation of body weight, food intake, and satiety. Expression of these genes involves different epigenetic processes, such as DNA methylation, histone modification, or non-coding micro-RNA synthesis, as well as variations in the gene sequence, which results in functional alterations. Evolutionary and non-evolutionary (i.e., genetic drift, migration, and founder’s effect) factors have shaped the genetic predisposition or protection from obesity in modern human populations. Understanding and knowing the pathogenesis of obesity will lead to prevention and treatment strategies not only for obesity, but also for other related diseases.

## 1. Introduction

Obesity represents a burden on public health. Its prevalence is increasing at an alarming rate worldwide, and it ranks fifth among causes of death worldwide [1,2,3,4].

The incidence of obesity has tripled in the last few decades, such that more than two thirds (70.2%) of the United States adult population is overweight or obese, and almost half of adults (48.5%) live with prediabetes or diabetes, conditions strictly linked to obesity. The prevalence of being overweight and obesity in adults in Europe is 34.8% and 12.8%, respectively [5,6]. The prevalence of being overweight and obesity in Europe is higher than that of North African children living in their own countries or as immigrants in Europe [7]. A recent analysis of data obtained in Italy from the Italian Barometer Obesity Report (https://ibdofoundation.com/, accessed on 12 May 2023) [8] estimates that over 25 million people are overweight in Italy, or more than 46 percent of adults (over 23 million people), and 26.3 per cent among children and adolescents aged three to seventeen (two million and two hundred thousand people).

There are several possible mechanisms leading to obesity. The traditional view is usually that the main cause is the significantly more excess energy stored than the energy the body used. The excess energy is stored in fat cells, thereby developing the characteristic obesity pathology [9]. However, the increasing obesity prevalence is due to a combination of individual factors, including genetics, epigenetics, metagenomics, as well as environmental, cultural, and behavioral factors [10]. Understanding and examining, in detail, all the factors active in rising rates is essential for the prevention of diseases associated with obesity, such as diabetes, cardiovascular disease, cancer, and many digestive diseases [11,12]. The obesity epidemic also has a major impact on the economy with its huge health care costs [12,13].

Genetics certainly play an important role in the genesis of the obesity phenotype. Just think that genetic variation between people accounts for 50 to 70% of the difference in BMI, but genetics are complex. The amount of body fat is affected by many different factors, including how efficiently the digestive system extracts nutrients from food, how easily nutrients are stored as fat or burned as fuel, and how hungry we feel. Each of these factors is influenced by hundreds of genes. The contribution of each of these genes is not defined. While there are some genetic variants that greatly increase the risk of obesity, these are rare. For most of us, each gene contributes only a small fraction of risk, but all together, they can mean the difference between being naturally thin and having to struggle to maintain a healthy weight. Even people with a similar risk of obesity may have different genetic reasons for that risk.

Because of this, different people may need different approaches to weight management. The heritability of obesity and body weight in general is high, and a small number of confirmed monogenic forms of obesity have been identified [14,15]. Genome-wide association studies (GWAS) have identified numerous genes associated with syndromic monogenic, non-syndromic monogenic, oligogenic, and polygenic obesity [16,17,18]. However, these genetic variants explain only a small proportion of the heritability of obesity [19]. The time course of the epidemic, which started only about 60–70 years ago, means that large-scale changes in the genetic makeup of the population are unlikely. As with all modern epidemics of noncommunicable diseases (NCDs), obesity is also due to the complex interactions between health, economic growth, and development associated with universal trends, such as population aging, indiscriminate urbanization, climate change, and the worldwide spread of unhealthy lifestyles. Therefore, the role of the environment is equally fundamental, and obesity is part of the list of evolutionary mismatch pathologies: the progressive selection, in times characterized by poor access to food, of genes capable of both optimizing energy storage and allowing the intake of a high number of calories when available while encouraging energy conservation by avoiding physical activities when not strictly necessary, makes, today, those who are carriers, more susceptible to weight gain.

We carried out an informal and non-systematic search by selecting analyses and genetic association studies to detect all possible clues suggesting the role of the genome in influencing the obesity phenotype and to find possible therapeutic strategies based on selective targets. We will try to analyze the contribution of the obesity susceptibility genes that we have been carrying around for millennia and how these only recently, in combination with the modern environment, make us obese.

## 2. Monogenic Obesity: Single Gene, Strong Effect

Monogenic forms of obesity are rare. These are caused by mutations in a single gene [17,20,21,22,23] (Table 1).

The most common monogenic form of obesity is caused by mutations in the melanocortin-4 receptor (*MC4R*) gene (OMIM*155541) [22,24]. *MC4R* is a G protein-coupled receptor that is critical in the leptin–melanocortin pathway. *MC4R* is expressed in districts, such as the hypothalamus, as well as in adipocytes, but its deficiency in mice leads to obesity with a framework that includes hyperphagia and hyperinsulinemia [25]. In humans, more than 200 distinct genetic variants are associated with early-onset obesity and hyperphagia [26]. MC4R-associated obesity is the most common monogenic form of obesity, with a reported prevalence of up to 6%. Interestingly, a recent report of the eMERGE III Network on 24,956 participants from 11 US sites has reported that ~7.3% of examined subjects carried at least one coding variant in the MC4R coding region [27].

Mutations in leptin and leptin receptor genes (*LEP*, OMIM*164160; *LEPR*, OMIM*601007) have been described since the end of the 1990s. They are considered to have biological roles related to the proteins encoded by these genes [28]. In fact, they regulate fat metabolism and energy intake. Leptin is mainly involved in the decrease in appetite [29]. Administration of recombinant leptin in individuals or mice leads to a decrease in food intake and body weight [30]. As a result of these studies, it had been thought that leptin could be used as well as insulin [31]. However, most obese people do not have lack of leptin, but instead hyperleptinemia, which leads to “leptin resistance” [32]. For this reason, caution is recommended in the administration of leptin to obese subjects who are not well characterized molecularly.

Among the recognized genes, some encode for components of the melanocortin pathway, such us *POMC* and PCSK1. *POMC* (gene encoding for Proopiomelanocortin, OMIM*176830) is an appetite inhibitory gene that produces peptides that regulates body weight, and, among them are α- and β-melanocyte stimulating hormone (MSH) [33,34]. Mutations in this gene lead to hyperphagia and obesity, both in mice and humans [35,36]. In patients with congenital POMC deficiency, there is now a pharmacologic alternative that counteracts hyperphagia and obesity. This therapy is based on a MC4R agonist, setmelanotide, that compensates for the lack of Melanocyte Stimulating Hormone (MSH) due to either mutations of the enzymes that cleave POMC or to LepR mutations [37]. In fact, *PCSK1* prohormone convertase 1 encodes for PC1 Proconvertase 1 (OMIM*162150), and it is involved in processing peptide hormones, such as POMC [38]. Studies on patients with severe obesity identified a mutation in the *PCSK1* gene [39,40]. In pediatric courts with malabsorptive diarrhea, there have been identified mutations in the *PCSK1* gene. Most of those patients have early-onset obesity and hyperphagia [41,42].

Another gene of interest is the *NPY* gene, which encodes neuropeptide Y (OMIM*162640), a potent hypothalamic orexigenic peptide [43]. There is an association between *the NPY* gene and obesity. In fact, administration of neuropeptide NPY causes increased food intake and obesity. Neuropeptide Y neurons inhibit POMC neurons that decrease appetite, and they activate orexin- and melanin-concentrating hormones, which are appetite-inducing peptides [44].

An Important recognized gene is the *FTO* gene (OMIM*610966), known as “the fat mass and obesity associated gene”, which is an obesity susceptibility gene [45]. However, the gene shows the strongest known effect on common types of obesity, despite some variants of the gene increasing obesity risk, while others lower it. Nevertheless, the effects of FTO depend on the environment. Even high-risk forms of FTO have little effect on body fat among people who obtain lots of exercise or eat low-fat diets. The association between *FTO* and obesity is documented in different populations [46,47,48]. *FTO* inactivation in mice leads to postnatal growth retardation and a decrease in adipose tissue. Those lean mice have an increase in energy expenditure and systemic sympathetic activation and a decrease in hyperphagia, which is interrelated with spontaneous locomotor activity [49]. Interestingly, researchers at the University College London Obesity Research Center have shown that the presence of a specific variant of the *FTO* gene, called rs9939609 A, correlates with a lower feeling of satiety following meals. AA individuals, homozygous for this form of the gene, in fact have altered circulating levels of the hormone ghrelin, a central element of the neuroendocrine control of energy homeostasis [50]. However, it is still unclear how FTO variants and, in particular, non-coding variants, act at the molecular level. It is possible that some of them form functional connections to *IRX3* and *IRX5* that are one Mb apart [51,52], suggesting that variants within FTO might also influence long-distance gene regulatory mechanisms. Interestingly, Sobreira et al. [53] examined chromatin interactions between key genes of the region where *FTO* maps, and they elegantly demonstrated that coding variants of *FTO* have pleiotropic effects across multiple tissues, whereas non-coding variants mediate their phenotypic effects by specific tissue and temporal regulation of gene expression. These results demonstrate that the genetic architecture of associated loci in this region associated with obesity may result in broad pleiotropy, allelic heterogeneity, shared allelic effects between tissues, and temporally limited effects.

Genes with high penetrance towards the obesity phenotype are also found in syndromic forms of obesity manifesting with additional phenotypes and often presenting as part of a distinct genetic syndrome [54] (Table 1). Obesity associated with syndromic forms is usually severe and has an early onset with neurodevelopmental disorders and/or polymalformative syndrome. Syndromic forms of obesity are generally due to genomic segment copy number (CNV) variants or consequence of chromosomal abnormalities [54]. It is estimated that there are at least eighty genetic syndromes in which obesity is considered a main feature. Among syndromic forms with clinical picture including obesity there is Prader-Willi syndrome, which occurs due to the lack of expression of the paternal genes of the chromosome region 15: 15q11.2–q13, and it is characterized by symptoms and signs such as developmental delay, intellectual disability, severe infant hypotonia, early-childhood onset obesity, and hyperphagia [55].

Some of the most serious complications of this syndrome are related precisely to obesity and hyperphagia [55,56]. It is worthwhile to note and observe how the proximal interstitial 6q deletion is associated with a syndromic form of obesity that closely resembles Prader-Willi syndrome. In addition to obesity, several other endocrinopathies are also found, such as hypothyroidism, growth hormone deficiency, and hypogonadotropic hypogonadism. The endocrine phenotype of interstitial deletion 6q remains largely unknown, although the clinical similarities between Prader-Willi syndrome and interstitial deletion 6q suggest that endocrine abnormalities may also contribute to the interstitial deletion of the 6q phenotype. In this region is mapped the Single-minded 1 (*SIM1* OMIM*603128) gene, which encodes a transcription factor active during neurogenesis, proposed as a candidate for involvement in certain dysmorphic features (particularly facial and cranial features), brain development abnormalities, and/or disability cognition of Down syndrome [57]. For this syndrome, there is no known therapy to date, but it is known that there is reduced hypothalamic expression in these patients, causing a deficiency of oxytocin. For this reason, it was hypothesized that the same might benefit from oxytocin treatment. This type of treatment could serve to increase the sense of satiety, providing energy intake and expenditure balance [58].

Studies also delve into fragile X syndrome due to the repetition of CGG triplets (over then 200), which results in the silencing of the *FMR1* gene (OMIM*309550). It is associated with retardation and phenotypic features, such as prominent ears and elongated face. In more than 30% of those affected, obesity problems are found [59]. In some patients, metformin has been tried—already used for patients with type 2 diabetes and for the treatment of obesity in children and adults—to improve circadian rhythms and restore memory deficits precisely because these problems can be explained in patients with fragile X by the dysregulation of insulin signaling. In patients treated with such therapy, there is not only behavioral and communicative improvement, but also hyperphagia and lower body weight [60,61].

Another less prevalent syndrome is WAGR syndrome, caused by 11p13 deletions, and about 30% of those affected have obesity. Most of them have a deletion in the *BDNF* gene (brain-derived neurotrophic factor) (OMIM*113505), suggesting that obesity phenotype is due to the alteration in this gene [62,63].

## 3. Polygenic Obesity: Multiple Genes, Small Effects

As with all complex and multifactorial traits, many susceptibility genes contribute to the obesity phenotype. Polygenic obesity is certainly the most common form of obesity, which inevitably results from the interaction between obesogenic environmental factors and variants of different risk alleles. Unlike monogenic and syndromic obesity, it is more difficult to study this variety of obesity because, hypothetically, every individual has a different number of variants, which can contribute to the phenotype with a different “weight” and with complex allelic interactions that are difficult to evaluate [64]. Numerous studies carried out in the last 15 years using the candidate-gene approach or the association through GWAS have led to the discovery of over 700 susceptibility SNPs in our genome [4,65] (Table 2).

One of the first obesity genes identified is FTO was described in monogenic form. Some genetic variants increase the risk of obesity, while others reduce it. Even the high-risk forms of FTO have little effect on body fat among people who exercise a lot or eat low-fat diets. FTO gene encodes a nuclear protein of the non-heme iron superfamily related to AlkB and 2-oxoglutarate-dependent oxygenase. Recently, FTO was shown to mediate m6A demethylation of long-spliced element 1 (LINE1) RNA in mouse embryonic stem cells (mESCs), by regulating LINE1 RNA abundance and local chromatin status, which, in turn, modulates the transcription of LINE1-containing genes. These results suggest broad effects of LINE1 RNA m6A demethylation by FTO in mammals [66].

Numerous SNPs identified as susceptibility genomic biomarkers for obesity are indeed found in more or less extensive phenotypes of obesity as BMI, adiposity traits, adipogenesis, lipid metabolism, insulin secretion, and genes involved in neuro-circuits of appetite and satiety, such as BDNF, NEGR, IRS1, and INSIG2 [65,67,68,69,70,71]. The identification of risk variants and, therefore, of the genes of which they are expression, is essential to ensure a better understanding of the pathogenesis of obesity by highlighting unknown molecular mechanisms. Consequently, new “targets” will be available against which to develop effective drugs. However, the simultaneous study of these SNPs will lead to the early identification of people with the highest risk of developing the obesity phenotype and associated pathologies, as well as to activate prevention programs (e.g., appropriate diet and drugs), which will allow, in the immediate future, to significantly reduce their risk of developing diseases related to obesity [72]. To date, there are no accurate genetic tests that allow the precise identification of subjects at risk of obesity and, therefore, their possible utility in clinical practice. The reason for this scarce clinical utility can be attributed precisely to the complex interaction between genetic and environmental factors, and, often, also to the randomness of the interactions that can arise during embryonic development or during postnatal life. In fact, the susceptibility alleles directly involved in risk assessment never act in an isolated and univocal way, but in harmony and often influence each other in their effects (epistasis) [73]. Interestingly, a recent study carried out through the analysis of a hospital cohort of over 30,000 individuals for whom electronic medical records, genetic, and lifestyle data were available, made it possible to demonstrate that the risk of obesity conferred by about a hundred common genetic variants was associated with a high BMI, and this risk was mitigated by a healthier lifestyle of the patient [74]. This study demonstrates how the genetic influence on obesity is associated not with a single variant, but rather with the complex interplay of dozens of genes, the cumulative effects of which put some patients at increased risk of developing the obesity phenotype and other pathologies, such as diabetes. For this reason, it is necessary to develop new algorithms to accurately determine the personalized polygenic risk scores (PRS) for everyone. However, developing sensitive and accurate PRS requires cohorts of over a million or more people to ensure we cover all phenotypes and endophenotypes associated with obesity.

Some considerations, however, can be made based on the GWAS results. In fact, looking at the data obtained from a very broad meta-analysis both in terms of subjects studied (almost 340,000) and of number of polymorphisms (SNPs), it needs to be underscored the fact that all of the 97 loci associated with BMI affect genes expressed at the level of the central nervous system [4]. Moreover, if we consider that all the monogenic obesities identified so far concern exclusively genes encoding hypothalamic proteins (except for leptin) mainly involved in the regulation of appetite, we can conclude that obesity is a neurobehavioral disease.

## 4. Epigenetics: The Dress That Genes Wear

Genes are not “naked” in our genome but are subject to chemical modification regulating gene activities [75,76]. This process includes DNA methylation, RNA-mediated processes, and histone modifications, which are some of the mechanisms included in epigenomics and regulate genomic stability and structure [77]. Some studies identified CpG sites associated with obesity and suggested that variability and methylation could predict obesity [78]. Other CpG sites were associated with BMI and waist circumference [79]. Epigenetic changes can be influenced by environmental factors, such as diet, physical activity, and exposure to toxins. Studies have shown that maternal obesity and gestational diabetes can lead to epigenetic changes in offspring, which can increase the risk of obesity and metabolic disorders later in life [77]. For this reason, it is crucial to identify and classify epigenetic marks on the genome of obese individuals to understand how each susceptibility gene is read to produce a distinct phenotype. This also provides a better explanation of how the environment plays a significant role in influencing how genes are expressed. Since 2013, several association studies have been performed to map the epigenome (EWAS) and understand the various expressions of genes in different tissues. These studies heralded a new era in the study of the genetics of obesity [80,81]. EWAS demonstrated that alteration in DNA methylation is most often a consequence of adiposity [82]. In studies conducted in obese children, genes such as *ABCC5, ARID1B*, *CD247*, *CHD3*, *CNTN1*, *CPNE6*, *EEFSEC*, *FAM53B*, *GABBR1*, *IGFBP6*, *KCNQ1*, *MAD1L1*, *RPS6KA2*, *SNO2*, *SH2B2*, *SLC43A1*, *SLCO3A1*, *STK40*, and *SYNJ2* are characterized by changes in DNA methylation [83,84]. Interestingly, some studies show how fetal under- and over-nutrition, regulated by maternal diet, are associated with an increased risk of obesity [85]. Individuals prenatally exposed to famine were at higher risk of becoming overweight [86,87]. People exposed to Dutch Hunger Winter have a lower degree of DNA methylation of the imprinted *IGF2* gene (Data obtained by comparing unexposed siblings) [86]. Mothers who suffered hunger show alteration in DNA methylation of genes that play a role in metabolic diseases, including *INSIGF2*, *GNASAS1*, *MEG3*, *IL10*, and *LEP* [88]. Other studies have detected, in overweight pregnant women, an increase in DNA methylation in four CpG sites (*MMP7*, *KCNK4*, *TRPM5*, and *NFKB1*) in cord blood DNA [89]. Offspring of obese and underweight mothers show different methylation patterns compared to the offspring of normal-weight mothers [90].

Micro-RNA (miRNA) dysregulation has also been observed in obesity [91]; miRNAs are short non-coding RNA molecules that post-transcriptionally repress gene expression by binding to untranslated regions and encoding target mRNA sequences. It has been demonstrated that miR-27a, miR-103, and miR-143 are upregulated in the adipose tissue of obese individuals. Furthermore, miR-122 has been implicated in the development of non-alcoholic fatty liver disease (NAFLD). Furthermore, several miRNAs are differentially expressed in obese individuals with insulin resistance versus those without insulin resistance [1]. miRNAs could be used as biomarkers of obesity and related disorders, but further validation and qualification research is needed to define them as reliable genomic biomarkers.

The study of the epigenetics of obesity is of particular interest for developing and activating prevention programs at the population level. Indeed, as stated by Danielle Reed, “Epigenetics is sort of like writing in pencil, whereas genetics is really writing in pen” [92]. This means that it is possible to intervene on social determinants and nutritional profiles, for example, to reduce obesity levels.

## 5. Genetics and Environment Therapies

It is conceivable to foresee that, in the coming years, genetic analysis technologies will identify obesity susceptibility genes, and PRS algorithms will be available on a large scale to identify individuals at high risk of developing obesity and related diseases from birth or before. A child with a predilection toward a genetic form of obesity may be treated with a particular diet, or they may be metabolically “reprogrammed” with the administration of drugs, hormones, and, perhaps, in some rare cases of monogenic obesity, with gene therapy. All this presupposes an accurate medical history and adequate genetic counseling before and after the test. A detailed family history, as well as psychosocial history, diet assessment, and physical activity/exercise are key elements in the diagnostic and therapeutic process of obesity. In fact, only after excluding endocrine causes of obesity and syndromic forms will it be possible to carry out genetic or epigenetic tests to identify adequate and actionable therapeutic targets. Identifying the genetic variants associated with diet-related diseases to study the variability of an individual’s response to diet and nutrition is now considered an interesting and innovative line of research [93]. Studies of the genetics-related effects of nutrition focus on specific gene and SNP variants and how they interact with dietary habits (nutrigenomics). Not only what we eat, but also when it becomes important for some individuals, is important. In fact, eating late has been linked to less weight loss in the AA genotype of *PLIN1* 14995 A > T carriers [94]. Another example is shown by how the best results of a low-fat diet were found in overweight and obese subjects with the *IRS1* rs2943641 CC genotype [95]. Another study indicated that overweight and obese individuals with the T allele of *PPM1K* rs1440581 have greater weight loss on a low-carbohydrate diet [96]. Additionally, a genetic variant in the *FGF21* region improves the risk of developing obesity, determining the preference for carbohydrate intake [97]. These studies demonstrate that there is an interaction between diet and genes that supports precision nutrition interventions that consider interindividual variability [98].

It is indisputable that food has a huge impact on mental and physical health. Genetic and biochemical knowledge today make the time ripe for clinical trials of specific approaches to the prevention or treatment of diseases, such as obesity, using food as medicine [99].

The Food and Drug Administration (FDA, Silver Spring, MD, USA) has approved two drugs intended for patients with genetic causes of obesity: metreleptin and setmelanotide. The other drugs, such as semaglutide, liraglutide, phentermine–topiramate, and naltrexone–bupropion is approved for weight loss in the general population and can be used to treat patients with genetic obesity. Metreleptin is a leptin analog used to treat patients with congenital generalized lipodystrophy in leptin-deficient patients with mutations in the leptin gene. However, metreleptin cannot be used in patients with leptin receptor mutations or mutations downstream of the leptin signaling pathway. The use of this drug is monitored by the FDA’s Risk Evaluation and Mitigation Strategies (REMS) Panel. Setmelanotide is a MC4R agonist used in obese patients with genetic mutations in the *POMC*, *PCSK1*, or *LEPR* genes and Bardet Biedl syndrome. The advantage of this drug is that it acts directly on the *MC4R* receptor, bypassing multiple targets, which may be mutated in the leptin pathway.

From what has been described above, it is, therefore, possible to state that distinct interventions of a nutritional or pharmacological type can modify the epigenome of the body for the benefit of patients with obesity. However, in some cases, it is a necessary recourse to resort to bariatric surgery. This, too, can determine epigenomic changes, such as the pattern of exosomal micro-RNAs of adipocytes, and it can cause epigenetic changes in differential methylated regions in *HOXB1*, *PRKCZ*, *SLC38A10*, and *SECTM1* genes [100].

Regarding surgical treatment, according to some studies, outcomes are worse in patients with Prader-Willi syndrome than in patients with common obesity [101]. According to other studies, the results obtained in patients undergoing gastrectomy and mini gastric bypass were the same in PWS patient and in the control group [101]. There are some studies on patients with monogenic obesity associated with alteration of the leptin/melanocortin pathway; the outcome of surgery in a patient with a homozygous variant in the LEPR gene showed that there is an initial weight loss maintained for six years, and then the patient returned to the obese form [102]. A large study was carried out by a Dutch group on patients, including 30 of whom who had mutations in genes such as *POMC* and *PCSK1,* with results not significantly different from noncarrier patients of these mutations [103]. An additional Chinese study analyzing patients carrying variants in the *LEP*, *LEPR*, *SIM1* and *PCSK1* genes demonstrated significantly less weight loss at six years than patients without the variant [104]. Concerning *MC4R* variants, studies show how there is an identical weight loss at one year for patients with the *MC4R* variants and the control group [105] or without significant differences after one and two years after RYGB (Roux-en-Y Gastric Bypass) [105]. Cooiman et al., after revisional RYGB after one and two years of follow-up, observed that *MC4R* patients have insufficient weight loss [103].

Even regular exercise can be considered an “environmental therapy”, as it can cause widespread changes in DNA methylation in the *RUNX1*, *NDUFC2*, *THADA*, *MEF2A*, and *PRKAA2* genes. Indeed, it has been shown that, in patients who lose weight, their methylation profiles of the *RYR1*, *TUBA3C,* and *BDNF* genes resemble those of lean individuals [77,85,100].

## 6. Outlook

A century ago, obesity was rare. Now, people around the world are gaining weight, with 70.2% of adults in the US currently being overweight or obese. Obesity is linked to increased rates of health problems, such as cardiovascular disease and diabetes. Why the sudden change? Is obesity in our genes or has something in our environment made us fat in recent decades? The answer is both. People vary in how easily they gain weight, and much of this variation is encoded in genes and passed down through families. However, the rise in obesity has occurred too rapidly to be caused by genetic changes. Our genes are the same as they were 10,000 years ago, and it is known that it takes at least 184–1840 generations of natural selection (that is about 5000 to 50,000 years) for a human population to undergo significant genetic change. As obesity rates have nearly tripled in just a few generations, the increase likely has more to do with changes to our environment and lifestyle than our genes [106]. Genetic variations related to obesity have been with us for millennia, but only in combination with the modern environment make us obese. This idea was first proposed by geneticist James Neel in 1962 [107], with the “thrifty genotype” hypothesis, suggesting that, in modern society, susceptibility to diseases, such as diabetes, may be a deleterious consequence, which had previously been beneficial in human ancestral environments [108]. However, although the parsimonious gene hypothesis proposed by James Neel nearly 50 years ago was a tempting idea, it has been criticized for being oversimplified and not fully supported by empirical evidence. An alternative hypothesis, the “gene drift” hypothesis, proposes that random genetic drift, rather than natural selection, may have led to the accumulation of genetic variants that increase the risk of obesity and related metabolic disorders in modern populations [109]. In support of this hypothesis, there would be the high prevalence of obesity in some geographically isolated populations, such as the Samoans, who have maintained genetic homogeneity and kept risk alleles at high concentrations due to the founder effect. In fact, GWAS studies have allowed the identification of risk variants strongly associated with BMI in the *CREBRF* gene [110,111,112]. It is possible that the selection of risk alleles as hypothesized by J. Neel could occur only for specific loci, as has been suggested by Southam and Coll. (2009) [113], but there are no consistent models of selection that provide conclusive confirmation of the thrifty genotype hypothesis. The common genetic variants that predispose to obesity found in different populations are uncommon. However, there are exceptions, such as the rs7566605 C allele of the *INSIG2* gene, present in 10% of individuals of different ethnic origins, suggesting that it is ancient and is consistent with the hypothesis that such alleles only became deleterious in modern times [70]. Similar results have also been suggested for the *FTO* and *TCF7L2* genes [113]. These results are not sufficient to support the notion of a universal mechanism to explain the high prevalence of type 2 diabetes and obesity. It is likely that, in the coming years, large population-based studies using NGS will lead to the identification of other common obesity susceptibility loci. In this sense, Akbari et al. [114], using whole exome sequencing, identified rare genetic variants affecting BMI in a sample of over 600,000 people from the UK, US, and Mexico. The authors identified genes in which rare non-synonymous variants were associated with higher or lower BMI, providing insight into the genetics underlying human adiposity. In vitro and in vivo experiments on animal models confirmed the “protective” role of the *GPR75* LOF variants against weight gain and the related disruption of glucose and insulin metabolism. This could suggest the development of inhibitors of GPR75 cellular functions as potential therapeutic drugs for obesity. Interestingly, functional SNPs within *GPR75* were found recently within the evolutionary constraint DNA regions, which represent 10.7% of the human genome, which is conserved in more than 98% of the 240 placental mammals analyzed [115].

Large-scale human exome sequencing, coupled with the analysis of methylation episignatures across the genome, is likely to become an increasingly important entry point for uncovering mechanistic insights into the biology of obesity in mammals such as humans.

In conclusion, obesity is a complex health problem influenced by various factors, including behavior, genetics, and the environment. Understanding the genetic effects on appetite regulation is crucial for effective treatment and prevention of obesity. Genetics play a significant role in the regulation of appetite and the development of obesity. Exploration of genetic factors and metabolic pathways is critical for the precise treatment and prevention of obesity.

## Figures and Tables

**Table 1:** Genes involved in monogenic obesity and in syndromic forms with an obese phenotype.

Gene Name and HGNC Approved Gene Symbol	OMIM	Biological Function
***MC4R*** **(MELANOCORTI 4 RECEPTOR)**	155541	Regulation of energy homeostasis
***LEP*** ***(LEPTIN)***	164160	Regulation of fat metabolism and food intake; decrease in appetite
***LEPR*** **(LEPTIN RECEPTOR)**	601007	Receptor for Leptin Involved in regulation of fat metabolism
***POMC*** ***(PROOPIOMELANOCORTIN)***	176830	Produces peptide that regulates body weight
***PCSK1*** **(PROPROTEIN CONVERTASE-1)**	162150	Involved in processing of prohormones (es. proopiomelanocortin) and other regulators of energy metabolism
***NPY*** **(NEUROPEPTIDE Y)**	162640	Regulation of neuronal activity stimulates food intake
**FTO** **(FAT MASS AND OBESITY ASSOCIATION GENE)**	610966	Regulation of fat mass, adipogenesis, and body weight
***SIM1*** ***(SIM bHLH TRANSCRIPTION FACTOR 1)***	603128	Pleiotropic effects during embryogenesis and in the adult; involved in neurogenesis
***FMR1*** ***(FRAGILE X MESSENGER RIBONUCLEOPROTEIN 1)***	309550	Neuronal development and synaptic plasticity
***BDNF*** **(BRAIN-DERIVED NEUROTROPHIC FACTOR)**	113505	Promotes the survival and differentiation of selected neuronal populations. Regulation of synaptic transmission and plasticity

**Table 2:** A selection of genes identified as candidates for obesity susceptibility.

Gene Name and HGNC Approved Gene Symbol	OMIM	Biological Function
***NEGR1*** ***(NEURONAL GROWTH REGULATOR 1)***	613173	Role in neural cell recognition and neurite outgrowth
***IRS1*** ***(INSULIN RECEPTOR SUBSTRATE 1)***	147545	Mediates cellular control by insulin
***INSIG2*** ***(INSULIN-INDUCED GENE 2)***	608660	Mediates feedback control of cholesterol synthesis
***ABCC5*** ***(ATP-BINDING CASSETTE, SUBFAMILY C, MEMBER 5)***	605251	Transport of endogenous metabolites ejects physiological compounds, and xenobiotics from cells
***ARID1B*** ***(AT-RICH INTERACTION DOMAIN-CONTAINING PROTEIN 1B)***	614556	Reshapes chromatin by which transcription of selected genes is activated or repressed
***CD247*** ***(CD247 ANTIGEN)***	186780	Role in adaptive immune response as a part of TCR-CD3 complex present on T-lymphocyte cell surface
***CHD3*** ***(CHROMODOMAIN HELICASE DNA-BINDING PROTEIN 3)***	602120	ATP-dependent helicase; it binds and distorts nucleosomal DNA
***CNTN1*** ***(CONTACTIN 1)***	600016	Involved in nervous system development
***CPNE6*** ***(COPINE VI)***	605688	Plays a role in calcium-mediated intracellular processes
***EEFSEC*** ***(EUKARYOTIC ELONGATION FACTOR, SELENOCYSTEINE-tRNA-SPECIFIC)***	607695	Necessary for the incorporation of selenocysteine into proteins
***FAM53B*** ***(FAMILY WITH SEQUENCE SIMILARITY 53, MEMBER B)***	617289	Involved in positive regulation of canonical Wnt signaling pathway
***GABBR1*** ***(GAMMA-AMINOBUTYRIC ACID B RECEPTOR 1)***	603540	Regulates proliferation and function of hematopoietic stem and progenitor cells
***IGFBP6*** ***(INSULIN-LIKE GROWTH FACTOR-BINDING PROTEIN 6)***	146735	Autocrine growth inhibitor
***KCNQ1*** ***(POTASSIUM CHANNEL, VOLTAGE-GATED, KQT-LIKE SUBFAMILY, MEMBER 1)***	607542	Encode for potassium channel
***MAD1L1*** ***(MITOTIC ARREST DEFICIENT 1 LIKE 1)***	602686	Component of the spindle-assembly checkpoint
***RPS6KA2*** ***(RIBOSOMAL PROTEIN S6 KINASE A2)***	601685	Regulates translation, mediates cellular proliferation, survival and differentiation
***SNO2- SKIL*** ***(ONCOGENE SNO)***	165340	May have regulatory role in cell division or differentiation in response to extracellular signals
***SH2B2*** ***(SH2B ADAPTOR PROTEIN 2)***	605300	Adapter protein; involved in multiple signaling pathways
***SLC43A1*** ***(SOLUTE CARRIER FAMILY 43 MEMBER 1)***	603733	Transport activity
***SLCO3A1*** ***(SOLUTE CARRIER ORGANIC ANION TRANSPORTER FAMILY, MEMBER 3A1)***	612435	Transport activity
***STK40*** ***(SERINE/THREONINE PROTEIN KINASE 40)***	609437	Inhibited activation of NFKB and p53
***SYNJ2*** ***(SYNAPTOJANIN 2)***	609410	Encodes for Inositol 5-phosphatase which may be involved in distinct membrane trafficking and signal transduction pathways
***GNAS-AS1*** ***(GNAS COMPLEX LOCUS, ANTISENSE TRANSCRIPT 1)***	610540	Produces a paternally imprinted antisense RNA transcript that helps regulate the GNAS complex locus
***MEG3*** ***(MATERNALLY EXPRESSED GENE 3)***	605636	Inhibits tumor cell proliferation in vitro; interacts with the tumor suppressor p53; regulates p53 target gene expression
***IL10*** ***(INTERLEUKIN 10)***	124092	Encodes for major immune regulatory cytokine.
***MMP7*** ***(MATRIX METALLOPROTEINASE 7)***	178990	Degrades casein, gelatins of types I, III, IV, and V, and fibronectin. Activates procollagenase
***KCNK4*** ***(POTASSIUM CHANNEL, SUBFAMILY K, MEMBER 4)***	605720	Encodes a member of the TWIK-related arachidonic acid-stimulated two pore potassium channel subfamily
***TRPM5*** ***(TRANSIENT RECEPTOR POTENTIAL CATION C)***	604600	Plays a central role in taste transduction
***NFKB1*** ***(NUCLEAR FACTOR KAPPA-B, SUBUNIT 1)***	164011	Regulates TLR-induced pro-inflammatory gene expression in activated macrophages; role in innate immune response
***PLIN1*** ***(PERILIPIN 1)***	170290	Modulator of adipocyte lipid metabolism
***PPM1K*** ***(PROTEIN PHOSPHATASE, MAGNESIUM/MANGANESE-DEPENDENT, 1K)***	611065	Essential for cellular survival and development
***FGF21*** ***(FIBROBLAST GROWTH FACTOR 21)***	609436	Regulates systemic glucose homeostasis and insulin sensitivity
***HOXB1*** ***(HOMEOBOX B1)***	142968	Transcription factor for developmental regulatory system
***PRKCZ*** ***(PROTEIN KINASE C, ZETA FORM)***	176982	Involved in a variety of cellular processes such as proliferation, differentiation, and secretion
***SLC38A10*** ***(SOLUTE CARRIER FAMILY 38 (AMINO ACID TRANSPORTER, MEMBER 10)***	616525	Transporter activity
***SECTM1*** ***(SECRETED AND TRANSMEMBRANE 1)***	602602	Encodes a transmembrane and secreted protein; thought to be involved in hematopoietic and/or immune system processes
***NDUFC2*** ***(NADH-UBIQUINONE OXIDOREDUCTASE SUBUNIT C2)***	603845	Encodes an accessory subunit of mitochondrial respiratory complex I
***THADA*** ***(THADA ARMADILLO REPEAT-CONTAINING PROTEIN)***	611800	Encoded protein is likely involved in the death receptor pathway and apoptosis
***MEF2A*** ***(MYOCYTE ENHANCER FACTOR 2A)***	600660	Activation of numerous growth factor- and stress-induced genes; control of cell growth, survival and apoptosis
***PRKAA2*** ***(ROTEIN KINASE, AMP-ACTIVATED, CATALYTIC, ALPHA-2)***	600497	Regulation of cellular energy metabolism
***RYR1*** ***(RYANODINE RECEPTOR 1)***	180901	Codifies for a calcium release channel
***TUBA3C*** ***(TUBULIN, ALPHA-3C)***	602528	Constitution of microtubules
***CREBRF*** ***(CREB3 RECRUITMENT FACTOR)***	617109	Regulates cell proliferation
***TCF7L2*** ***(TRANSCRIPTION FACTOR 7-LIKE 2)***	602228	Participates in the Wnt signaling pathway
***GPR75*** ***(G PROTEIN-COUPLED RECEPTOR 75)***	606704	May play a role in neuron survival May regulate insulin secretion by pancreatic islet cells

## Data Availability

Not applicable.
